# A Potential Role for JAK Inhibitors in Refractory Photoaggravated Atopic Dermatitis

**DOI:** 10.1111/phpp.70067

**Published:** 2025-12-06

**Authors:** John Warner‐Levy, Layal Khoursheed, Donna Parkin, Jean Ayer

**Affiliations:** ^1^ Faculty of Biology, Medicine and Health The University of Manchester Manchester UK; ^2^ Centre for Dermatology Research The University of Manchester & Salford Royal NHS Foundation Trust Manchester UK; ^3^ Manchester Academic Health Science Centre Manchester UK

1

Photoaggravated atopic dermatitis (PAD) is an uncommon subtype of atopic dermatitis (AD), characterised by symptom exacerbation in response to ultraviolet (UV) radiation [1, 2]. While the IL‐4/IL‐13 inhibitor dupilumab is the standard systemic therapy for moderate‐to‐severe AD, its effectiveness within the context of PAD is unclear, with evidence limited to isolated case reports [3]. JAK inhibitors, which target several immune pathways implicated in AD [4], may offer a promising alternative for individuals diagnosed with PAD. However, to date, no studies have systematically evaluated JAK inhibitors in this setting. Here, we present preliminary data indicating that JAK inhibitors may provide benefits over dupilumab in managing refractory PAD.

This retrospective cohort study involved an observational analysis of patients treated according to standard care protocols and represents an expanded analysis of previously published work [5]. Recruitment spanned from November 1, 2018, to September 30, 2024 with initial immunosuppressive therapy consisting of either methotrexate or cyclosporine. Participants transferring to dupilumab were followed at 16 weeks, while those switching to JAK inhibitors were assessed at 12 weeks. Data were collected between November 2024 and January 2025. Eligibility required participants to be ≥ 18 years with PAD refractory to initial immunosuppressive therapy. Exclusion criteria included concurrent systemic immunosuppressive treatments other than dupilumab or JAK inhibitors, and the presence of other photosensitive dermatoses. Outcomes included changes in safety profiles, clinical severity scores (EASI, POEM, DLQI), and abnormal phototesting thresholds. Interventions comprised subcutaneous dupilumab, starting with a 600 mg loading dose followed by 300 mg every 2 weeks, and oral JAK inhibitors, including upadacitinib (15 mg daily), baricitinib (4 mg daily) and abrocitinib (100 mg daily). Ethical approval was waived as all interventions were routine clinical care, with the study conducted in accordance with the Declaration of Helsinki.

Ten patients satisfied the inclusion criteria and were included in the study. One participant received sequential therapy and was thus assigned to both treatment groups. This resulted in six participants treated with dupilumab (mean age 42.3 ± 8.5 years; 2 males, 4 females) and five treated with a JAK inhibitor (mean age 42.6 ± 13.7 years; 4 males, 1 female). Among the participants treated with JAK inhibitors, three received upadacitinib, and one each received baricitinib and abrocitinib. Baseline monochromator phototesting data were unavailable for one participant in the JAK inhibitor group, leaving four participants in the JAK inhibitor group and six in the dupilumab group available for phototesting analysis.

As shown in Figure 1, JAK inhibitors demonstrated a greater reduction in the proportion of participants demonstrating abnormal phototesting thresholds compared to dupilumab. Treatment with JAK inhibitors also yielded more pronounced improvements in clinical severity scores, with greater reductions from baseline observed in both POEM (71.6% vs. 56.7%) and EASI (78.4% vs. 61.4%) scores. In contrast, reductions in DLQI, both weekly (86.2% vs. 83.6%) and yearly (49.1% vs. 50.9%), were comparable between the two treatments. No serious adverse events were reported in either group. Underlying data can be found in Tables S1 and S2.

**FIGURE 1:**
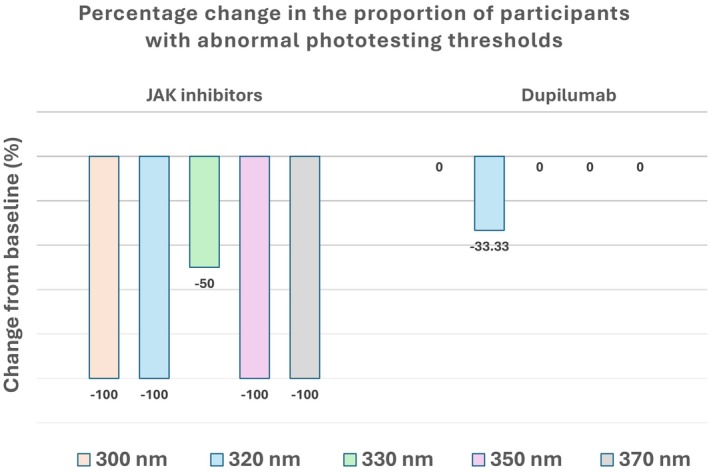
Percentage change in the proportion of participants with abnormal phototesting thresholds.

The role of JAK/STAT signalling in AD has been described [4], and along with patient data [6], supports both biologic and clinical plausibility for the use of JAK inhibitors in PAD. Emerging evidence indicates that following solar‐simulated UV exposure, individuals diagnosed with PAD exhibit early activation of innate immune responses, as well as Th1 and Th22 pathways, followed by a predominant Th2 response [7]. Although IL‐13 upregulation is reported [7], the broader immunomodulatory effects of JAK inhibitors may better address the immune dysregulation specific to PAD, compared to the more selective IL‐4/IL‐13 inhibition provided by dupilumab [3]. Additional research is needed to explore how differences in JAK inhibitor selectivity influence efficacy.

Though these findings represent the first known evidence demonstrating the superiority of JAK inhibitors over dupilumab in managing refractory PAD, limitations include a small study population, albeit unavoidable due to the rarity of the disease, a short follow‐up period, and a retrospective design. Finally, although EASI, POEM, and DLQI are validated measures of global disease burden, they lack the ability to assess severity within specific disease regions, highlighting a potential avenue for future research and tool development. Importantly, the findings warrant prospective validation through multicentre studies, which are essential not only to validate the safety and efficacy of JAK inhibitors in PAD but also to evaluate their potential as a first‐line therapeutic option.

## Funding

The authors have nothing to report.

## Conflicts of Interest

The authors declare no conflicts of interest.

## Supporting information


**Table S1:** Abnormal phototesting thresholds at baseline.
**Table S2:** Pre‐ and post‐treatment clinical severity scores.

## Data Availability

The data that support the findings of this study are available from the corresponding author upon reasonable request.
