# Supplementary material for: A Potential Role for JAK Inhibitors in Refractory Photoaggravated Atopic Dermatitis
Source: Photodermatol Photoimmunol Photomed. 2025 Dec 6;42(1):e70067. doi: 10.1111/phpp.70067 (PMC12683711; doi:10.1111/phpp.70067)
Supplement: Supplementary file 1 — Table S1: Abnormal phototesting thresholds at baseline. Table S2: Pre‐ and post‐treatment clinical severity scores. [file PHPP-42-e70067-s001.docx]

*Supplementary Table One: Abnormal phototesting thresholds at baseline*

| Wavelength | JAK inhibitor participants with abnormal phototesting at baseline (n=4) | Dupilumab participants with abnormal phototesting at baseline (n=6) |
| --- | --- | --- |
| *300 nm* | 2 (50%) | 3 (50%) |
| *320 nm* | 1 (25%) | 3 (50%) |
| *330 nm* | 2 (50%) | 1 (17%) |
| *350 nm* | 1 (25%) | 1 (17%) |
| *370 nm* | 3 (75%) | 1 (17%) |

*Supplementary Table Two: Pre- and post-treatment clinical severity scores*

|  | JAK inhibitors (n=5) | Dupilumab (n=6) |
| --- | --- | --- |
| *Mean pre-treatment clinical severity scores (SD)* |  |  |
| DLQI week | 17.4 (4.3) | 19.3 (6.8) |
| DLQI year | 21.2 (3.3) | 25.8 (5.2) |
| POEM | 17.6 (3.1) | 25.0 (4.0) |
| EASI | 32.2 (12.5) | 27.0 (6.6) |
| *Mean post-treatment clinical severity scores (SD)* |  |  |
| DLQI week | 2.4 (3.0) | 3.2 (2.9) |
| DLQI year | 10.8 (8.6) | 12.7 (9.1) |
| POEM | 5.0 (2.1) | 10.8 (9.9) |
| EASI | 7.0 (0.6) | 10.5 (4.3) |
